# Dimensions of Musical Creativity

**DOI:** 10.3389/fnins.2020.578932

**Published:** 2020-11-30

**Authors:** Andrea Schiavio, Mathias Benedek

**Affiliations:** ^1^Centre for Systematic Musicology, University of Graz, Graz, Austria; ^2^Institute of Psychology, University of Graz, Graz, Austria

**Keywords:** musical creativity, creative cognition, music performance, music composition, enactive cognition

## Abstract

Current literature on creative cognition has developed rich conceptual landscapes dedicated to the analysis of both individual and collective forms of creativity. This work has favored the emergence of unifying theories on domain-general creative abilities in which the main experiential, behavioral, computational, and neural aspects involved in everyday creativity are examined and discussed. But while such accounts have gained important analytical leverage for describing the overall conditions and mechanisms through which creativity emerges and operates, they necessarily leave contextual forms of creativity less explored. Among the latter, musical practices have recently drawn the attention of scholars interested in its creative properties as well as in the creative potential of those who engage with them. In the present article, we compare previously posed theories of creativity in musical and non-musical domains to lay the basis of a conceptual framework that mitigates the tension between (i) individual and collective and (ii) domain-general and domain-specific perspectives on creativity. In doing so, we draw from a range of scholarship in music and enactive cognitive science, and propose that creative cognition may be best understood as a process of skillful organism–environment adaptation that one cultivates endlessly. With its focus on embodiment, plurality, and adaptiveness, our account points to a structured unity between living systems and their world, disclosing a variety of novel analytical resources for research and theory across different dimensions of (musical) creativity.

The proliferation of novel enquiries, theories, and methodologies emerging within a research domain, often gives rise to a multiplicity of sub-areas exhibiting narrower focus and increased specialization. Explorations within specialized fields can facilitate insights on very specific aspects of a problem, which sometimes only apply in this very context, and sometimes bear relevance to overarching issues. Hence, the process of fragmentation poses the fascinating challenge of whether findings observed in resulting sub-fields generalize across them, and how they could be fruitfully integrated to expand their explanatory reach. By bringing together insights from complementary as well as contrasting schools of thought, such integrative accounts usually appear well-positioned to offer richer understandings of the range of phenomena under examination. In the natural sciences, for example, the synthesis of diverse theories into generally accepted canons has been often associated with increased knowledge and scientific improvement. Balietti et al. (2015) illustrate this point by referring to Newton’s blending of “celestial and terrestrial forces” and to how “Maxwell unified electricity and magnetism in one single force called electromagnetism.”

Notably, the tendency to build on narrower lines of enquiry to develop broader frameworks is not limited to the natural sciences; it also arises in much research on human cognition and its various manifestations. Here, a number of key concepts, such as “experience,” “thought,” or “consciousness,” have been traditionally addressed from a variety of angles, leading to approaches that employ different analytical instruments ranging from the examination of one’s neural activity to the classification of verbal reports and descriptions. As such, a number of objects of investigation in this area remain blurred and ill-understood; and unlike phenomena with more specific, measurable features, the properties associated with mind and subjectivity do not easily fit within one domain, purportedly leaking into other scholarly territories. Perhaps the potential ambiguity of outcomes that this process brings forth is a price well worth paying for promoting dialogue and epistemological diversity. And in any case, whether the process of systematization will give rise to a unified “grand” theory or not, heterogeneity of ideas can be generally considered as a sign of good health for scientific enterprise.

In this regard, the study of creativity is no exception. In fact, the latter can be seen as emblematic for the potential conflicts that arise when considerations from a wide spectrum of research trajectories are combined into novel constructs, methods, and theoretical models. Indeed, while plurality of approaches is a valuable aspect of scientific discovery and its conventions of significance (see e.g., Benedek and Jauk, 2014), “we should also consider how each one of them constructs the meaning of creativity and guides its practice” (Glãveanu, 2014, p. 6). Within the rich variety of voices populating the creativity discourse, we highlight two distinctions that are particularly prominent and that have fragmented, if not polarized the field. The first one involves the notions of *individual* and *collective* creativities. As we will see, this differentiation refers to two perspectives that conceive of creativity as a property of the lone agent and as a multiply realized, social phenomenon, respectively. The second important distinction involves viewing creativity from a *domain-general* or *domain-specific* perspective. Both distinctions, we suggest, highlight specific fragmentations in the field, as scholars usually tend to adhere to either approach and pursue it predominantly in their research.

In the present article, we take a closer look at both distinctions. We present individual *vs*. collective as well as domain-general *vs*. domain-specific accounts of creativity, and review relevant contributions that adhere to such perspectives. Because our aim is to mitigate tensions between said approaches, in turn laying down the basis of a framework that looks at creativity in more synergetic terms, we subsequently explore scholarly domains in which these dichotomies appear being less rigid. We begin with examples, arguments, and intuitions from the areas of music performance and music composition. Our analysis emphasizes how individual and collective forms of creativity may not be understood as alternatives: recent music scholarship trades the focus on single agents and groups to their underlying relational principles and embodied entanglements, helping us re-organize the conceptual topography of creative phenomena (see Reybrouck, 2006; Nagy, 2017; Cook, 2018; van der Schyff et al., 2018). We then introduce the main tenets of *enactive cognitive science*—a school of thought that conceives of the mind as situated action-as-perception (Varela et al., 1991; Di Paolo et al., 2017; Gallagher, 2017). We observe how, on this account, two main properties of creativity (*novelty* and *functionality*) can also be seen to play an enabling role in shaping mental life more generally, describing the capacity of biological systems to establish, transform, and re-organize meaningful adaptive relationships with their niche. This helps us trace a continuum between general bio-cognitive principles and creative thought and action, thereby reframing the issue of domain-general vs. domain-specific creativity into more conciliatory terms. In doing so, we offer an understanding of creativity as a process of skillful organism–world adaptation. This interpretation allows us to move beyond the study of explicit thinking abilities that characterizes much creativity research to include more situated, dynamic, and world-involving aspects of cognitive life and subjectivity, which may not be captured when postulating initial distinctions. Finally, we proceed to illustrate how this conceptual framework may lead to precise empirical questions by outlining a possible experimental paradigm. Figure 1 depicts the main structure of the paper and its main points.

**FIGURE 1:**
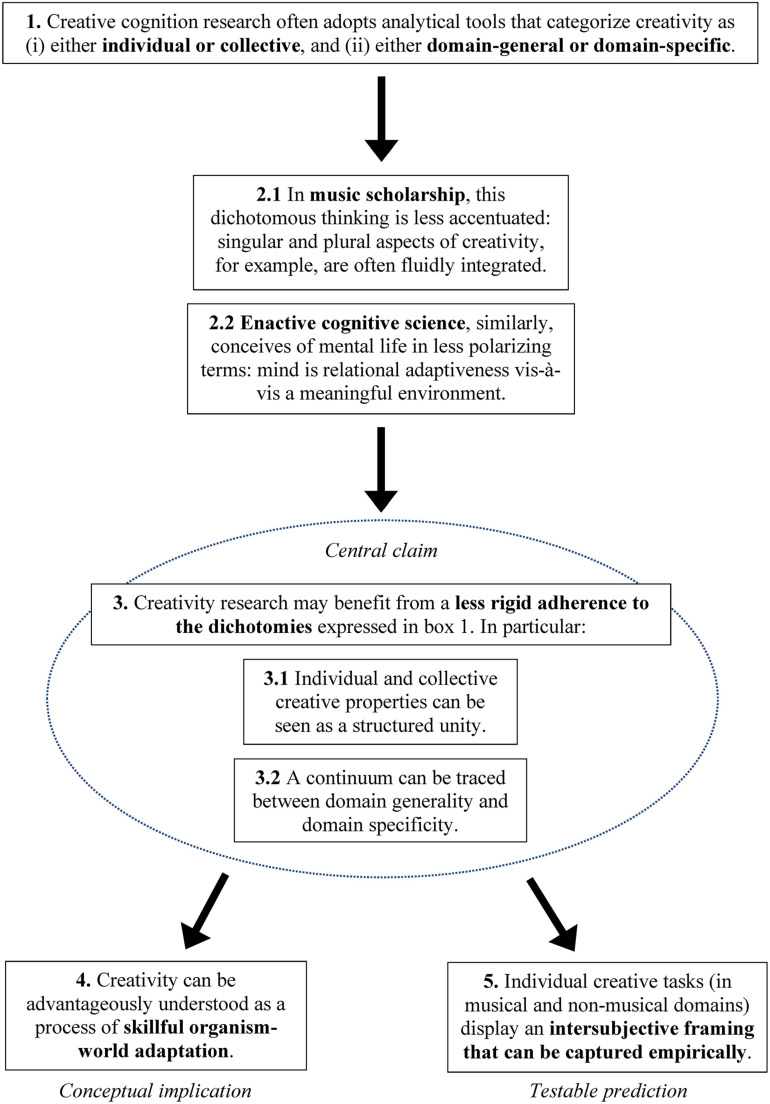
Our contribution moves from the presentation and critical assessment of previously posed theories in creative cognition research (box 1). This is followed by a novel interpretation of recent findings from the domain of music, and by an analysis of the main tenets of enactive cognitive science (2, 2.1, 2.2). This leads us to the central claim of the paper, which is presented in box 3, and articulated in two sub-claims (3.1, 3.2). The major conceptual implication of the proposal appears in box 4, whereas box 5 introduces the main idea of a possible experimental setting, which is described in detail in the conclusive section of the paper.

Before we begin, it should be noted that several authors who work in embodied and enactive cognitive science, as well as ecological dynamics and distributed cognition, have written on creative processes (see e.g., Hristovski et al., 2011; Vallee-Tourangeau and Vallee-Tourangeau, 2014; Vallee-Tourangeau et al., 2016; Kimmel et al., 2018; Torrance and Schumann, 2019). However, as Malinin (2019) argues “there is [still] minimal evidence of embodied cognition approaches in creativity research or pedagogical practices for teaching creativity skills.” This paper, therefore, builds on this scholarship to provide additional grounding to such lines of research, stimulating a dialogue between different perspectives on creativity in music and beyond. To do so, we employ an interdisciplinary approach that brings together humanities, performance studies, and neuroscience in multiple ways, generating hypotheses and insights relevant to scholars belonging to each of these areas. In the next section, we start this enterprise by presenting a number of core concepts at the heart of creativity research, and we associate them with perspectives looking at creativity as a phenomenon that is either (i) individual or collective or (ii) domain-specific or domain-general.

## Creative Cognition

Work on individual creativity and collective creativity, as well as research taking a domain-general or domain-specific perspective, has provided important advances to our understanding of creative thought and discovery. In this section, we offer an overview of the main tenets and findings from each of these approaches, exploring differences and lines of continuity between them. Here, we do not intend to provide a comprehensive review of the field; instead, we wish to introduce a number of key contributions that (explicitly or implicitly) tend to adhere to one or both dichotomies. This overview thus serves a double function: on the one hand, it outlines what advantages and limitations emerge when the study of creativity is framed within such given perspectives; on the other hand, it provides general insights that set the stage for later, more specific, observations.

### An Individual Perspective on Creativity

Creativity research taking an individual perspective aims to understand how and why a person is creative. What happens in the mind and brain of this person when she generates a creative idea or produces a piece of creative work? What cognitive factors participate in driving creative thoughts and action? And why do some people have more creative ideas or accomplishments than others? In general, two types of creative thinking are commonly distinguished: creative idea generation and creative problem solving, also called “divergent” and “convergent” thinking creativity, with reference to Guilford’s structure of intellect model (Guilford, 1967). Creative idea generation refers to the production of different possible responses to ill-defined problems. While such open-ended problems have a large solution space, ideas will differ considerably in their creative quality, with some being more novel and effective than others. In contrast, creative problem solving (or insight problem solving) refers to finding a single correct solution to a problem that cannot be solved in a straightforward, analytical way. The process of solving such problems requires to reframe the problem representation in order to overcome predominant, but inadequate solution approaches, and is often accompanied by sudden experiences of insight.

The individual perspective is particularly well suited to examine the temporal dynamics of creativity (for a review, see Lubart, 2001). Relevant stages of creative problem solving commonly include preparation (i.e., engaging with a problem), incubation (i.e., when we are no longer consciously engaged with a problem, subconscious processing typically goes on), illumination (i.e., the moment of spontaneous insight as a potential solution comes to our mind), and verification (i.e., conscious evaluation of the candidate solution; see Wallas, 1926). Engagement in creative activity has also been associated with a state of flow, which is characterized by deep immersion and subjective feelings of ease and timelessness (Csikszentmihalyi, 1997).

Many empirical approaches have been developed to enable the study of divergent and convergent thinking creativity in standardized settings. A prominent example of a divergent thinking task is the “alternate uses” task, which asks participants to find creative new uses for common objects, such as a brick or car tires. Other popular divergent thinking tasks require experimental subjects to imagine consequences of utopian situations, suggest product improvements, complete abstract figures, or produce creative metaphors, as well as humorous puns. Performances in divergent thinking tasks are usually scored with respect to quantitative and qualitative aspects. Quantitative scoring assesses the total number of responses (i.e., ideational fluency) or the number of responses from different categories (i.e., ideational flexibility) produced in a given time. Creative quality, conversely, is commonly evaluated by raters, tabulated norms, or analyses of statistical infrequency (Barbot et al., 2019; Reiter-Palmon et al., 2019). There is broad consensus that a creative idea has to be both novel/unusual and effective/task-appropriate (Runco and Jaeger, 2012; Diedrich et al., 2015). Popular convergent thinking creativity tasks include the Remote Associates Test (Mednick, 1962), which asks to find a word that links three unrelated words, and various insight problems, such as the nine-dot problem (Gilhooly and Murphy, 2005). Besides divergent and convergent thinking creativity, also more complex creative production tasks are employed, asking to create drawings, write stories, or improvise on a musical instrument. Performances on these tasks are usually assessed by a panel of competent judges (i.e., consensual assessment technique; see Amabile, 1982). Reviews of the cognitive and neuroscience literature (Forgeard and Kaufman, 2016; Benedek et al., 2019) showed that most research explores creative idea generation (>50%), whereas less work investigates creative problem solving (10–20%) and product-based creativity (20–30%).

The availability of standardized measures of creative thinking enabled the investigation of the specific cognitive and brain processes underlying creative cognition, such as memory, control, and attention. The role of cognitive control in creative cognition has been a vexing problem as there is evidence for the relevance of both controlled, goal-directed, and spontaneous, undirected processes (for reviews, see Chrysikou, 2018; Benedek and Jauk, 2019). While active creative thinking benefits from effective strategies and high cognitive capacity, spontaneous processes may be particularly relevant for more complex creative work that runs into impasses and involves incubation phases (for dual process accounts of creative cognition, see Sowden et al., 2015; Benedek and Jauk, 2018). Neuroscience research has begun to shed light on the neural basis of creative cognition, which heavily relied on functional MRI (fMRI) studies on musical improvisation (Bengtsson et al., 2007; Berkowitz and Ansari, 2008; Limb and Braun, 2008; de Manzano and Ullén, 2012; Pinho et al., 2015; for a review, see Beaty, 2015). Musical improvisation was found to implicate brain regions of the executive-control network (ECN) and the default mode network (DMN). These networks were further shown to exhibit increased functional coupling not only during piano improvisation (Pinho et al., 2015) but also in poetry composition (Liu et al., 2015) and divergent thinking (Beaty et al., 2015). The ECN is typically involved in top-down control, whereas the DMN is mainly implicated in self-generated thought, which can be spontaneous as in mind-wandering, or goal-directed as in mental navigation (Andrews-Hanna et al., 2014; Christoff et al., 2016). The coupling of these large-scale brain networks during creative cognition is thought to reflect an interplay between controlled, evaluative and more undirected, generative processes (Beaty et al., 2016; Zabelina and Andrews-Hanna, 2016). Additionally, the salience network (SN), which is considered to be implicated in the dynamic transitions between DMN and ECN (Uddin, 2015), may contribute to creative thought by forwarding candidate ideas originating from the DMN to the ECN for high-order processing, such as idea evaluation (Beaty et al., 2015). A recent study has demonstrated that creative people have the ability to simultaneously engage these large-scale brain networks (Beaty et al., 2018a), suggesting that individual differences in the ability to simultaneously engage DMN, ECN, and SN regions can be viewed as a neurophysiological marker of creativity (for reviews, see Beaty et al., 2016, 2019).

The role of memory in creative cognition is similarly fascinating, as creative thinking is known to build on memory and yet must go beyond recall in order to create something new. Creative thought has been conceived of as a fruitful recombination of remote associative elements (Mednick, 1962), but it is an ongoing debate to what extent it relies on a more effective access to memory and/or a deviant organization of memory (Kenett et al., 2014; Benedek et al., 2017a). Neuroscience research revealed that both semantic memory and episodic memory play a chief role for creative cognition (e.g., Fink et al., 2015; Madore et al., 2015). It is important to note that episodic remembering represents a reconstructive process, and that there is increasing evidence that episodic memory networks (overlapping with the DMN) are also recruited during future thinking and creativity (Beaty et al., 2018b, 2020; for a review, see Schacter et al., 2012). Still, the generation of creative new ideas slightly differs from the recall of known original ideas in additionally recruiting the left anterior inferior parietal cortex, which again points to the involvement of executive processes for integrating memory content in new ways and supporting executively demanding mental simulations (Benedek et al., 2014b, 2018).

Creative cognition has been further variably associated with broad, leaky, defocused or focused attention (Zabelina, 2018). There is at least some consensus though that imagination involves internally directed attention. When we imagine something new, indeed, we usually ignore or suppress irrelevant sensory input (for a review, see Benedek, 2018). This internal focus of attention has a clear neurophysiological signature as evidenced by eye-behavior changes reflecting perceptual decoupling and visual disengagement (Annerer-Walcher et al., 2018), increased electroencephalogram (EEG) alpha activity especially in the frontal and right parietal regions (Benedek et al., 2014), and reduced visual network activity paired with increased right parietal brain activation (Benedek et al., 2016). Increases of EEG alpha activity are a particularly robust finding in creativity research (Lustenberger et al., 2015; Luft et al., 2018; Agnoli et al., 2020; for reviews, see Fink and Benedek, 2014; Stevens and Zabelina, 2019), representing inhibition of task-irrelevant (sensory) processing (Jensen et al., 2012; Klimesch, 2012), which appears crucial for sustained internally directed activities involving imagination and mental simulation. Indeed, musicians were found to exhibit increased frontal upper alpha-band activity during musical improvisation compared with rote playback (Lopata et al., 2017); in contrast, more accurate learning of new musical structures was associated with lower alpha power, potentially suggesting that less internal focus is necessary when retrieving more automatized procedures (Zioga et al., 2020). Musical learning was also associated with increased amplitude of relevant event-related potentials (ERPs; for a review of early EEG/ERP findings, see Dietrich and Kanso, 2010).

In all, creative thinking is increasingly understood in terms of a specific configuration of underlying memory, control, and attention processes and their neural substrates (Jung and Vartanian, 2018; Benedek and Fink, 2019). This set of neurocognitive functions generally endows people with the capacity to engage in creative thinking. Yet, people still differ considerably in their creative task performance and creative life-time accomplishments. It is the central mission of individual differences research of creativity to explore the range and reasons of this variability and to understand how differences in creative potential eventually lead to differences in real-life creative achievements. Available models assume that creative achievement relies, on the one hand, on the cognitive potential to think creatively and, on the other hand, on conative factors, such as personality, expertise, and environmental conditions (Amabile, 1983; Eysenck, 1995). Creative personality is associated with high openness to new experiences (Feist, 1998) as well as high intrinsic motivation to engage in creative behaviors (Benedek et al., 2020a). Beyond what has been traditionally labeled as everyday creativity, more professional forms of creativity crucially rely on high domain-specific expertise (Weisberg, 1993; Boden, 2004; Kaufman and Beghetto, 2009): one must know the tools and rules of a given domain very well to extend, re-develop, or eventually break them in creative ways. Research has also identified environmental factors that are conducive to creativity including stimulating others, supportive structures, or general *zeitgeist* (Amabile, 1983; Simonton, 1999). In the next section, we will present research that goes beyond the individual perspective introduced here to explore creativity at a group, or system, level.

### A Collective Perspective on Creativity

While we sometimes associate creativity with eccentric scientists or lone composers who withdraw themselves from society until their work is done, creativity is not always understood as a solitary activity. In fact, complex creative work typically relies on collaboration between experts from different fields, and creative performances often require an ensemble or team of contributors. Moreover, creative work develops and exists in the wider context of its sociocultural environment and specifically its recipients (Glãveanu, 2014). The effects of such an ecological dimension have been acknowledged in relevant theories of creativity: the four P model (Rhodes, 1961) speaks of *press* (referring to the relationship between creative agents and their environment), besides person, process, and product; the five A-model (Glãveanu, 2013) speaks of *audience*, besides actor, action, artifact, and affordances; and many other models highlight how creative activity takes place within, and is shaped by, its social and organizational settings (e.g., Amabile, 1982; Eysenck, 1995; Amabile and Pratt, 2016).

The empirical study of group creativity has traditionally looked at how creators interact, how different social conditions affect creative outcomes, and how people judge the creative work of others. Much attention has been devoted to the investigation of creative idea generation in groups (aka brainstorming). Brainstorming was thought to boost creative performance by harnessing the power of cognitive stimulation and increased motivation when people interact (Osborn, 1963). Closer investigation, however, revealed that groups often perform poorer than nominal groups (i.e., an equal number of individuals performing tasks individually yields higher total performance; Diehl and Stroebe, 1987). Different cognitive, affective, and motivational process losses have been identified to occur when people generate ideas together, including production blocking (i.e., idea generation is blocked for all but the one who speaks), evaluation apprehension, pressure for conformity, and free-riding tendencies (Pinsonneault et al., 1999; Dugosh and Paulus, 2005). Idea generation in groups thus involves process gains and losses, and better outcomes have been associated with moderate group sizes and a balance between individual and interactive performances, such as those realized in brainwriting and electronic brainstorming (De Rosa et al., 2007).

Creative behavior is also affected by the attitude and feedback of others. While creativity is generally viewed as desired and needed, people often tend to reject novel, creative ideas due to their unfamiliarity and uncertainty (Mueller et al., 2012). In a similar way, it has been shown that teachers usually value creativity in students, but do have reservations when working with students who show creative traits, such as non-conformity and disagreeableness (Scott, 1999). Creativity further relies on the intrinsic motivation to generate and on creative self-concept (Amabile, 1985; see also Karwowski and Kaufman, 2017); yet, extrinsic factors, such as creativity-contingent positive and task-focused performance feedback, can also support creative performance (Byron and Khazanchi, 2012). Inflated praise, however, can have adverse effects, such as encouraging to go for low-hanging fruits instead of meeting challenges that involve higher risks (Brummelman et al., 2014).

As human behavior is always embedded in a social context, creative activity is clearly shaped by social dynamics, usually in the form of explicit interactions and implicit expectations by others. Along these lines, a number of scholars now maintain that because self and society (i.e., individuals and groups, living systems, and environments) form a structured unity, any attempt to decouple its constitutive elements may give rise to only partial representations of the creative phenomenon (Gergen, 1994; Montuori and Purser, 1999; Sawyer, 1999). Here, further insights arise from attempts to reconcile individual and collective elements in multimodal approaches (Amabile, 1996). As we will see later on, music and musical practices are good examples of this. In this field, individuality and collectivity are often seen as complementary aspects of one’s musical life, providing together more than just a sum of their respective domains (see also Olivetti Belardinelli, 2002). This may have crucial implications for our understanding of musical creativity and for creative cognition more generally. Before engaging with this issue in more detail, however, we first wish to illustrate the rationale behind the second dichotomy we have previously individuated—that between domain-general and domain-specific perspectives on creativity.

### Domain-Generality, Domain-Specificity, and Music

Creativity can be observed in diverse contexts and at various levels of professionalism: designing a decoration or a spaceship, improvising in the kitchen or on stage—all are thought to rely on creativity. The large diversity of creative behaviors has motivated researchers to organize them into different boxes. On a quantitative level, a distinction is usually made between little-c, pro-c, and big-c creativities, which refer to everyday, professional, and eminent creativities, respectively (Kaufman and Beghetto, 2009). On a qualitative level, creative behaviors have been sorted into different creative domains. As Sternberg argues, however, “[t]he greatest challenge in understanding the domain-generality vs. specificity of creativity is in understanding the concept of a domain itself” (Sternberg, 2009, p. 25). Creativity tests are mostly distinguished into verbal and figural tests and less frequently include numerical and musical tests, based on its response modality (see Torrance, 1974, 1984). A closer examination of the neurocognitive processes involved in task performance, however, suggests that this distinction is not fully valid (Benedek and Fink, 2019). For example, the alternate uses task, a popular divergent thinking task, requires to find and write down creative uses of everyday objects and thus is considered a verbal task. Process analyses of task performance have revealed that a commonly adopted strategy is to mentally disassemble the object and create novel products from its parts (Gilhooly et al., 2007), which requires a visual representation of the object and mental simulation of how its parts can be meaningfully reassembled. The solution could actually be drawn as well, but providing verbal responses simply appears most convenient. Neuroscience research has offered further evidence that “verbal” creativity tasks substantially implicate visual and motor regions (Benedek et al., 2020b; Matheson and Kenett, 2020), suggesting that creative task performance relies on multimodal capacities. Hence, the classification of tasks by their response modality may tell us a little more than how ideas are expressed in the final step of ideation (Benedek, 2018), but it does not adequately capture the complexity of the underlying neurocognitive processes. Given the available evidence, and the highly associative, multimodal organization of our brain, it could even be questioned whether there exists something like a pure verbal or visual creativity task, challenging narrow conceptions of task domains.

Quite different classifications of creative domains are used at the level of creative behavior and achievement. A very basic distinction can be made between arts and sciences (Feist, 1998). More fine-grained categorizations consider several domains, such as literature, music, visual arts, performing arts, culinary arts, humor, architecture, as well as creativity in business, sports, sciences, or social contexts (Carson et al., 2005; Abraham, 2018; Diedrich et al., 2018). These domains attempt to capture the most common creative behaviors, and follow established organizations of education tracks and professions relevant to creativity. However, any domain classification will fall short to comprehensively cover and represent the vast range and ideocracies of creative expression. It is in the very nature of creativity to extend established structures, and creativity thrives most when boundaries are crossed (Shmailov, 2016). Task modalities and creative domains should thus not be understood as natural entities, rather, they point to certain conceptual differences that may prove useful to organize thought and research, highlighting the diversity of creative behavior in general.

These considerations well exemplify the distinction between domain-general and domain-specific views on creativity (Baer, 2015; Barbot and Tinio, 2015). The problem gravitates around the question of whether creativity observed across different domains relies on common cognitive resources or rather on different specialized capacities. Put simply, “the theory that creativity is domain-general […] predicts positive correlations among the levels of creativity exhibited by individuals in different domains. Domain specificity predicts the opposite” (Baer, 2012, p. 19). The latter conception may imply that we may need not just one, but many theories that examine creative thinking and behavior in different contexts. A promising candidate for a domain-general aspect of creativity is divergent thinking. As mentioned earlier, the latter refers to the process of coming up with creative ideas, which appears fundamental to all forms of creative expression. In fact, there is substantial evidence that divergent thinking ability plays a role for various domain-specific forms of creativity. Divergent thinking ability was shown to predict the creativity of humor production besides intelligence (Kellner and Benedek, 2017), as well as mathematical creativity besides mathematical competence (Schoevers et al., 2018). Divergent thinking ability also predicts creative life-time achievements assessed by self-reports across domains, especially when estimating latent correlations (Plucker, 1999; Jauk et al., 2014). Studies focusing on specific domains reported that divergent thinking ability predicted the level of creative accomplishments in advertisers (Agnoli et al., 2019) and the quality of improvisations in jazz students (Beaty et al., 2013), and was higher in professional dancers than in novices (Fink et al., 2009). Divergent thinking ability even distinguished between subdomains, as evidenced by higher creative potential in jazz musicians than in folk musicians (Benedek et al., 2014a). Other studies, however, found no relationship between divergent thinking ability and domain-specific creative accomplishments in a domain (e.g., film artists; Benedek et al., 2017b), which could partly be explained by the fact that highly accomplished artists sometimes show little compliance to participate in psychological tests of creativity. Further evidence comes from the analysis of self-reports of creativity. These self-reports tend to correlate substantially with people’s self-concept of creativity (Kaufman and Baer, 2004). Similarly, latent-class analyses of self-reported accomplishments revealed that people differ in the level of creativity rather than in creative domains (Silvia et al., 2009). These findings are consistent with the domain-general view of creativity, but it needs to be noted that they relied on convenience samples, such as university students who commonly do not exhibit very high levels of creative achievement.

The domain-specific view of creativity is typically supported by noting that relationships between divergent thinking ability and creative accomplishments are very low (Baer, 2015). Moreover, it is generally questioned whether eminent creative people, such as Albert Einstein, would have been equally successful in other domains, such as poetry (Kaufman and Baer, 2004). These questions are hard to test empirically, but many creative geniuses have in fact been polymaths, and there is evidence especially for a fruitful relationship between engagement in arts and scientific success (Root-Bernstein et al., 2008). Yet, it appears undisputed that the role of domain-specific expertise increases with more professional levels of creativity (Kaufman and Beghetto, 2009). Arguably, a person without any training in a given field (e.g., medicine, violin performance, etc.) will not be able to make substantial contributions to her respective field. From a domain-general perspective, the question remains whether a person with poor creative abilities could ever make substantial creative contributions to any area.

How do these deliberations apply to musical creativity? Generally speaking, music has fascinated neurocognitive research because “playing, listening to, and creating music involve practically every cognitive function” (Zatorre, 2005, p. 312), and it is often associated with strong emotions and experiences (Gabrielsson, 2001; Jäncke, 2008). Musical practices have also recently drawn the attention of scholars interested in their creative properties, as well as in the creative potential of those who engage with them, giving rise to a large number of interdisciplinary contributions situated at the crossroads of musicology, cognitive (neuro)science, as well as sociological and psychological research (see e.g., Burnard, 2012; Donin and Traube, 2016; Clarke and Doffman, 2017; Cook, 2018). And indeed, music is among the most popular domains in inventories of creative achievement (Diedrich et al., 2018). Interestingly, measures of creative cognitive potential do not really cover musical expression. There have been approaches to assess the potential for musical creativity in terms of basic abilities to generate novel melodies or rhythms in non-musicians (Berkowitz and Ansari, 2008), but it is more common to study musical creativity in professionals and in the moment-to-moment realization of their artistic outcomes. In the next section, we pick up a related thread as we focus on the contexts of music performance and music composition. Our aim is to critically engage with existing research and theory, assess a number of empirical findings, and show how individual and collective forms of creativity can be synergistically integrated. Among other things, we offer a novel interpretation of the results from an fMRI study by Lu et al. (2015). More specifically, we suggest that because the body of work we discuss in the following lines treats singular and plural creative dynamics in a flexible way, it challenges more static views that often characterize current creativity research.

## Musical Creativity Beyond *Solo* and *Tutti*

The study of musical creativity offers a good example of a research avenue that increasingly looks beyond the polarization of individual and collective perspectives to embrace a more unitary view—one that sees singular and plural dimensions of creative cognition as two sides of the same coin. Additionally, because music involves a vast range of culturally relevant experiences, behaviors, products, and entanglements, it constitutes an ideal field of enquiry to look at both discrete and wide creative competences: while musical practices are specific enough to be characterized by precise norms and conventions across different social contexts, they also disclose a theoretically unlimited variety of possibilities to extend existing artistic knowledge. Musical activities, as we will see more in detail later on, are also associated with a range of general cognitive capacities, making the analysis of domain-specificity and domain-generality particularly fascinating. The present section addresses these and other insights within two main musical areas taken as exemplary domains: performance and composition.

### Performing Music

When thinking about creative musical performance, probably the first thing that comes to mind is an improvising jazz ensemble (see e.g., Johnson-Laird, 1988; Sawyer, 1992; Bailey, 1993; Berliner, 1994; Wilson and MacDonald, 2017). It is easy to imagine group members engaged in free improvisation or taking turns to produce subtle expressive nuances while repeating the main theme, collaboratively changing tempo, accents, and beats, and developing melodic, harmonic, and timbric mutations. Expert improvisers, indeed, are known to transform performance into a process of mutual discovery and negotiation, where different motor, communicative, and imaginative parameters are dynamically generated, assembled, hybridized, and re-deployed to serve novel functions and guide their activity through known and unknown (musical) territories (see Murray, 1998; Doffman, 2009; Duby, 2018; Kimmel and Rogler, 2018; Kimmel et al., 2018; van der Schyff, 2019).

Seminal research by Sawyer (2003, 2006), among others, placed major emphasis on the emerging dynamics involved in the generation of creative action when groups of individuals cooperate. Specifically focusing on jazz musicians and artists devoted to improvisational practices, Sawyer conceived of interaction itself as the main locus of creativity. As reported by van der Schyff et al. (2018), the latter in such contexts (i) displays an unpredictable outcome, (ii) involves a moment-to-moment contingency where each person’s action depends on the one just before, (iii) remains based on an interactional effect where any given behavior can be changed by the activity of other participants, and (iv) is intrinsically collaborative (Sawyer and De Zutter, 2009, p. 82). Notably, such insights do not only apply to (joint) improvisational settings; they are also relevant to broader situations in which musical interaction unfolds at different levels and timescales. To gain a richer understanding of how these considerations may be applied to concrete musical contexts, in what follows, we present cases involving online and offline adaptations between composers and performers, joint musicking, and instances of solo music-making. This can help us develop a constructive dialogue between theoretical insights and real-life musical practices, showing how individual and collective creative dynamics can be strongly intermixed. The florid interplay of solo and group aspects in creative music-making that emerges from this discussion also anticipates later comparisons between domain-general and domain-specific creativities^1^ and motivates the testable hypothesis we present in the conclusive section.

For now, let us begin with a comparison between the verbal communication occurring between musicians, composers, or improvisers when planning, rehearsing, optimizing, or simply sharing information about a novel piece or performance (see e.g., Clarke et al., 2013, 2016; Biasutti, 2015, 2018) and the online patterns of non-verbal interaction and self-regulation exhibited by members of classical ensembles (see e.g., Davidson and Good, 2002; Biasutti et al., 2016). In both cases, outcomes can be hardly predicted with precision: complaints or suggestions voiced by instrumentalists regarding particularly complex musical configurations, for example, may change the composer’s initial plans in various ways, giving rise to a series of adaptive, constructive dialogues, in which a middle ground between the composer’s expressive needs and the performative constraints indicated by the performer is generally reached^2^ (see Doffman and Calvin, 2017; Whittall, 2017). Importantly, members of a music ensemble executing a piece (e.g., from the Western classical repertoire) are also subject to constant adaptive changes. As reported by Bishop (2018), co-players often employ anticipatory strategies to keep various musical parameters, under control thereby optimizing their joint performance (see also Bishop et al., 2013). An EEG study by Loehr et al. (2013), for example, showed that expert pianists can selectively monitor their own actions and those of their partner, anticipating individual and combined musical outcomes. Along these lines, Badino et al. (2014) quantitatively examined *via* Granger causality^3^ the coordination dynamics of string quartet members during normal and perturbed conditions, finding that more demanding musical passages necessitate more reciprocal interaction and mutual influence from the performers than less challenging sections. Singular and plural factors of performance, on this view, must be continuously monitored, transformed, and negotiated in a process of adaptation and mutual interaction.

Working collaborations between composers and performers, as well as online interactions within groups of musicians, illustrate well the spectrum of reciprocal dependencies involved in music-making. For example, performers and composers can cooperate to explore a particularly innovative solution by creatively re-defining the horizon of opportunities for action of a musical instrument: strings can be untuned, pianos can be “prepared,” tools and technologies can be adapted for various expressive necessities, and so forth. This can lead the interactors to challenge each other, build on their expertise, and develop novel creative synergies to redirect individual plans toward different outcomes (Sawyer, 2003). With regard to the online interactions within a performing ensemble, a further example may help. Consider here the cascade of changes and adaptations that even a simple shift in a musical parameter may give rise to: imagine how a rock band playing their most famous song during a live show may unintentionally slow down the chorus to facilitate the audience singing along, thus impacting the coordination dynamics between group members. Because availability of visual cues facilitates interaction and successful synchronization among co-performers (see Bishop and Goebl, 2015, 2018), musicians might move across the stage more than expected to optimize their visual communication. This unpredicted change of plans might disrupt the fluidity of their execution (as well as the visual impact of their live performance) particularly during the lead guitarist’s solo occurring after the chorus: away from her multi-function pedalboard, she could not use her favorite effect (say, wah-wah). To compensate for this loss, the bass player, so the story goes, decides to accompany the solo with unexpected high notes, generating new fascinating counterpoints on the spur of the moment. This vignette resonates with early insights from Jane Davidson, who maintains that “if the performer senses the many cues of the live performance context and interprets them positively, a new state of psychological awareness can be achieved which allows the individual to become both highly task-focused and able to explore spontaneous thoughts and feelings in a creative manner” (Davidson, 2002, p. 149).

More in general, these examples are offered to situate the initial insights on improvisation within a broader understanding of performative creativity as an adaptive phenomenon that plays out in situation of online and offline collaborations. In such contexts, one can observe a continuous interplay of individual and collective decisions, plans, memories, choices, feelings, behaviors, and musical ideas, and how these can be recursively re-organized and adapted at both personal and multi-personal level. This well aligns with work on creative thinking that explores the deep connections between control, memory, and attention (Benedek et al., 2016), highlighting the social side of these categories.

Remarkably, there is also an important sense in which these considerations speak to situations where subjects make music alone, by themselves. Indeed, recent work in the field has highlighted the compenetration of solo and joint aspects of musical practice, suggesting that individual settings are, in fact, intrinsically collaborative (see e.g., Høffding and Satne, 2019; see also Cuffari et al., 2015 for similar insights developed with respect to language). This work provides an apt counterpoint to research that focuses on more explicitly interactive creativity–where collective outcomes are conceived of as emergent properties of the joint effort of collaborating agents–and complements existing studies that engage with lone individuals and their solitary creative achievements (e.g., solo improvisation). Looking for “traces” of intersubjectivity within solo musical contexts, accordingly, could reveal how individual activity might be understood as inherently participatory, shedding in turn new light upon both solo and plural forms of performative experience and their creative manifestations (see also Frith, 1996; Folkestad, 2012; Loaiza, 2016; Cook, 2018). Albeit not generalizable, qualitative data recently collected with expert and novice musicians (Schiavio et al., under review) indicate that playing music in isolation often involves a felt presence of others based on the creative re-enactment of a shared repertoire of practices or an anticipated experience of music-making in context. The latter refers to situations in which “virtual others” are mentally constructed or imagined by solo performers (e.g., when rehearsing at home a piece for orchestra); the former condition, in which a social presence is reported to be perceived in solo musicking, is more difficult to address. Perhaps, it could be argued that adopting certain instrumental techniques while improvising, realizing an ornament on the flute when interpreting a baroque piece, or choosing a tempo where not explicitly indicated in the score reflects an already intersubjective structure constituted by a community of practice (see Wenger, 2002)—a product of a historically sedimented creative work to which one skillfully adapts. In other words, individual musical choices and solutions are here understood as part of broader cultural, historical, and technical milieux and therefore never fully independent from their social components (see again Høffding and Satne, 2019).

In the target study (Schiavio et al., under review), two broad categories were considered: agency and creativity. Interviewed participants referred to agency (i.e., the subjective feeling that one is the author of her own actions) by describing various bodily and emotional aspects central to their musical experience, and how they may involve a sense of shared corporeality even in cases of solo practice. To provide an example, consider the following quote from an expert singer: “I always try to be as close as possible to the original intentions of the composers. This puts me in a weird place because then I must account my emotions, my sensitivity, and my fingers. It is like, I can look at the world with the eyes of the composer, but still within my own body.” This self-other negotiation can also play out in more intuitive terms, and is further recognized by an expert pianist as follows: “when I rehearse by myself I can feel the composer and his intentions, yeah. I say ‘feel’ because there are no main thoughts here.” The same focus on intersubjectivity emerged when subjects were asked about creativity. The latter was associated with terms such as “adaptation,” “mutual connection,” or “a need to communicate with someone.” For instance, one novice stated that “creativity is linked to how I express myself, my body language, more than just making music. It is about interacting with who is around and who will eventually get in contact with what I sing and how.” This study provides rich descriptions of situations in which *prima facie* “solo” creativity is associated with a more socially relevant dimension. As hinted above, this also refers to “those mutually constitutive relationships through which, as they grow older together, [people] continually participate in each other’s coming-into-being” (Ingold and Hallam, 2007, p. 6, quoted in Cook, 2018, p. 9). There is thus a complex web of social factors involved in seemingly isolated musical practices^4^, which permeates creative and expressive musical outcomes of individuals (and groups).

The concrete cases of music-making we examined in this section (ranging from solo improvisation to ensemble performance) provide good examples of this broad network of factors shaping creative efforts. In the next section, we further unpack these insights and explore the adaptive interplay of individual and collective creativities in the context of music composition.

### Composing Music

Creative artifacts usually take form of aesthetically rewarding products, which carefully integrate original and familiar factors in various ways. As we have suggested in the previous section, music performers can often achieve such a goal by engaging in processes of interpersonal adaptation and discovery even when playing alone. In doing so, musicians creatively negotiate (consciously or unconsciously; see Simonton, 1988; Sawyer, 1992) manifold cognitive strategies to optimize their musicking, in a constant interaction with the community of practice in which they are embedded. By exploring these strategies at different (e.g., cultural, behavioral, neural, analytical) levels, the study of musical performance–understood as a visible process of (co-)creation–can contribute a novel perspective on the collision of individual and collective factors in creative activity.

In this section, we extend these insights to the domain of music composition, starting from cases where clear-cut distinctions between performers and composers may be too static to capture important aspects of their creative effort. We then discuss more traditional examples of (score-based) compositional practices drawing on recent empirical work that looks at both qualitative and neuro-functional data, pointing again to an overlap of singular and plural dynamics. This, importantly, includes both (i) creative products and (ii) creative processes. Regarding the former, it should be noted that musical outputs are usually evaluated: whether they are generated in isolation or with others, creative forms, ideas, or contents need other people to be assessed, judged, examined, and culturally located. Indeed, “[c]reativity has a property that is not true of all psychological constructs—it exists in the interaction of the stimulus and the beholder. A maker may view his or her work as creative, but if there is not an audience that sees it that way, the maker aside, then the work is not considered creative” (Sternberg and Kaufman, 2010, p. 468). Similarly, the association of solo and joint dimensions emerges in the processes of music-making when the repertoire of actions, choices, and musical ideas at the basis of musical creation is contextualized and historically situated: as Dillon (2006) reports, with reference to Amabile (1985) and Csikszentmihalyi (1988), the social framing of creative effort involves a dialectic process of negotiation where individuals, groups, and sedimented practices form a uniquely recursive structure, often problematizing issues that go beyond the analysis of psychological processes, such as those pertaining to copyright and artistic appropriation.

The shifting constraints and goals of musical performance, thus, invite explorations and induce variabilities that are crucial for music-making (e.g., musicians deliberately inhibiting or reinforcing control and focus) and reflect larger social and cultural dynamics involving fine-tuning of musical ideas and adaptations to existing practices and repertoires (e.g., how to interpret a piece in a historically informed way without simply reproducing the score). Moreover, because repertoires and musical conventions are collectively constructed over the years by an evolving community of practitioners^5^, there is a strong sense in which even individual creative musical actions emerge from, and embody, such a web of relationalities. This insight prompts us to rethink the traditionally stark differentiation–probably advocated among others by Schönberg and Stravinsky–between originators of genuine musical ideas (i.e., composers) and mere executors (i.e., performers), helping problematize the “authorial identity” of the formers (Cook, 2001, 2006). Consider the following quote from classical guitarist Pepe Romero:

“As a player, when you take a piece of music you have to feel and become in tune with that composer, with his mind and with his soul, and unite it to your own mind, to your own soul, to your own heart. Then you can recreate the music so it has a freshness, and it sounds when the player plays it like he is composing it also. Together [the composer and the player] make one and they merge together; you cannot tell where one begins and the other ends. I know that when I play, and the music is really flowing, I cannot tell the difference between the composer and myself” (quoted in Dobrian, 1991).

We have already seen how composers and instrumentalists often combine divergent and convergent thinking when collaborating, for example, when exploring together multiple musical possibilities to optimize a planned performance, and evaluating all alternatives through analysis, trials, and processes of mutual adaptations (see Webster, 1987; Wong and Lim, 2017). However, the quote above points to a more intimate synergy, which plays out during the act of musicking. While this context-dependent “fusion” between composer and performer reminds of situations of improvised or vernacular musical contexts, in which “the power relationships among those taking part are diffuse, uncentralized; all will have some authority and bear some responsibility” (Small, 1998, p. 115), it also runs deep in Western classical settings. Consider, for example, how the *re-creation* of a musical score through interpretation becomes a legitimate *creative* process when it involves an artistically significant, innovative output—a feature that has been somehow downplayed by more traditional accounts:

“[M]usic affords an apparently unlimited variety of interpretive options, and we could be much more adventurous in our exploration of them if our thinking about performance was more flexible. The idea of music as sounded writing gives rise to what […] I call the paradigm of reproduction: performance is seen as reproducing the work, or the structures embodied in the work, or the conditions of its early performances, or the intentions of its composer. Different as these formulations are–and the last can serve as a justification for almost anything–they all have one thing in common: no space is left for the creativity of performers”

(Cook, 2013, p. 3).

Given this emerging overlap of roles, one could wonder whether the recognition of performers as creators would somehow downplay the creative authority of composers. Data from another recent qualitative study (Schiavio et al., 2020) indicate that Western classical composers are generally well aware of the relational dynamics involved in their “solitary” creative effort. While there has been some resistance to adopt this methodology to explore creativity in composers and musicians (Juslin, 2019, pp. 31–32), we maintain that a first-person approach has the advantage to offer unique insights into their lived experience, providing concrete descriptions grounded in the respondents’ everyday musical activities. Comparably to performers, composers seem to benefit from the florid mixture of individuality and collectivity in generating creative ideas, referring to three inter-related aspects of their compositional experience: (i) the instantiation of an adaptive dialogue between themselves and their social and cultural environments (e.g., composers from the past, future audience, performers who will play the piece they are composing, etc.), (ii) the importance of an explorative drive informing their practice, and (iii), the physicality of their musical activity, that is, how body and action take part in shaping creative ideas and outcomes, particularly when directed toward specific musical instruments. In all, this may help us cast a new light on what internally directed attention entails in similar activities. Rather than a lack of focus on external information, it rather requires a continuous integration of internal and external dynamics, and involves what Nagy refers to as a “constant, parallel evolution of both creative awareness and activeness” (2017, p. 34). This decenters the creative locus from the individual to a uniquely developing organism–world system (more on this below).

The ranges of responses collected in this qualitative work also partly align with a recent fMRI study with 17 music composers conducted by Lu et al. (2015). Here, the researchers compared the participants’ functional networks during an imaginative compositional task (after looking at a page with one written bar of music) with resting states (measured before the task). Two main results were found: during the composing period, participants exhibited a decreased functional connectivity between visual and motor areas and a stronger functional connectivity between the anterior cingulate cortex (ACC) and the DMN. The authors discuss the former result in terms of instrument-specificity: all composers were asked to create music for an instrument they did not know how to play (i.e., the Chinese Zheng); the second result, instead, suggests a context-dependent integration of emotional, combinative, and evaluative processes sub-serving how participants mentally manipulated sounds to convey emotions. We could speculate that this latter outcome also points to a “hidden” social dimension: sub-regions of the ACC (particularly its dorsal component) exhibit functions involved in the detection and appraisal of socially oriented (e.g., emotional) information (see e.g., Behrens et al., 2009; Apps et al., 2016), complementing existing evidence that implicates the ACC in the adaptation and monitoring of online motor activity (see Hochman et al., 2014; Mado Proverbio, 2019). Similarly, the class of midline and lateral cortical areas known as DMN (Andrews-Hanna et al., 2010, 2014)–whose activity has been usually associated with both mind-wandering states (Gould van Praag et al., 2017) and self-focused attention (Raichle et al., 2001)–“has been shown to play a critical role in various aspects of human social behavior” (Saris et al., 2020). In particular:

“The medial temporal DMN subsystem is associated with recollection of experiences and autobiographical processing, and is comprised of the hippocampal formation, retrosplenial cortex, inferior parietal lobule, and ventromedial PFC [prefrontal cortex] […]. The dorsal medial DMN subsystem, on the other hand, is predominantly involved in socially colored, meta-cognitive processes and mentalizing (i.e., inferences about others’ internal state)”

(Saris et al., 2020).

As Bashwiner (2018) notes, there is already a relatively long tradition postulating a direct correlation between DMN and divergent thinking, and therefore its implication in music-related generative ideation is not surprising (see also Beaty, 2015; Bashwiner et al., 2016; Beaty et al., 2016). With this in mind, considering both theoretical arguments and empirical data, the conjecture can be advanced that individual creative ideation in music composition reflects wider social dynamics, involving multiple neural substrates dedicated to the integration of intra-personal and inter-personal information^6^.

Work in isolation, moreover, is only one manifestation of how composers create music. We have seen already how they often collaborate with performers to optimize given plans and jointly (re-)adapt musical intuitions and forms. Composer Luciano Berio, for example, admits that the “first *Sequenza* […] was composed in 1958 for the flute of Severino Gazzelloni, and it wasn’t certainly a case that in these years we were together in Darmstadt, as it wasn’t a case that [for the other Sequenze] I have met the Harp of Francis Pierre, and […] the voice of Cathy Barberian” (Berio, 1981, p. 97, *translated from Italian*). In fact, there are many practices, experiences, and behaviors associated with composing music. These range from the systematic application of mathematical principles (sometimes adopted in contemporary Western classical music) to the creative impulse of young children and infants, who extend their natural curiosity to the world of sounds and progressively organize and develop their sonic discoveries in a deliberate way^7^ (see Schiavio et al., 2017). In pedagogical settings, as Burnard (2006) reminds us, research often adopts psychometric assessments of creative musical thinking (e.g., Webster, 1992; Hickey, 1995, 2000), as well as ratings of children’s musical compositions (Webster and Hickey, 1995; Hickey, 1997) in both individual and collaborative settings. Another example is collaborative songwriting in adults—where teams of composers are assembled to collaboratively create music, particularly pop songs (Bennett, 2012). This last case resonates well with approaches inspired by sociocultural and ethnomusicological insights, where the tangible result of creative doings is often thought to involve different (and sometimes invisible) hands. An understanding of musical creativity as a multiply-realized, adaptive phenomenon, however, does not entail a sole focus on groups, or explicitly collective forms of creative activity: music ensembles are formed by individuals who constantly negotiate meanings and bring forth their personal goals, emotions, and motivations, during performance or composition. Similarly, “an overemphasis on collective composition […] ran the danger of mystifying creative processes into myth and making invisible the creative contributions of individuals” (Hill, 2018, p. 100).

Instead, our analysis highlights the fluid integration of Persons (creators), Processes (thoughts and actions), Products (artifacts), and Press (cultural contingencies) in the creative musical moment (Rhodes, 1961). Musicians operate and generate artistic outputs in a living culture where solo and joint dimensions are tightly related and often hardly distinguishable. Accordingly, we have examined how individual and collective perspectives are intertwined in cases of score re-creation (i.e., by performers) and offered examples of more canonical acts of music composition (i.e., work in isolation) displaying intrinsically social components. The material discussed in this section points to an understanding of creative musical practice as a process of continuous, adaptive negotiation between individual and collective factors. This suggests that a research strategy that posits an initial distinction between these two levels might be necessarily limited. In what is next, we ground these insights into a broader framework–that of enactive cognitive science–and explore the links between adaptiveness, creativity, and mental life more generally.

## The Ubiquity of Skillful Adaptation

In this section, we examine what enactive cognitive science can offer to creativity research, with particular regard to the issue of domain-generality *vs*. domain-specificity. We begin by recognizing that not only does skillful adaptation play a crucial role in creative musical practice (as we saw above); instead, it also enables the development of more general organism–world couplings—a basic bio-cognitive capacity that characterizes living systems of different degrees of complexity. We individuate two important features of such couplings: *functionality* and *novelty*. These latter, on this view, are thought to lie at the same time at the heart of creative cognition (Runco and Jaeger, 2012) and of mental life more generally; in both cases, they contribute to the construction and maintenance of meaningful relationships between living systems and their environment in which local and global dynamics are fluidly integrated. We conclude that strong differentiations between domain-general and domain-specific creative activities cannot be drawn with accuracy. Said differently, we argue that (i) what we usually describe as domain-specific creative effort relies on a more general tendency to establish novel and functional relationships with the world; (ii) but because the various concrete manifestations of such a tendency (the patterns of adaptations enacted by each living system, the value and significance from which such couplings originate and contribute to develop, etc.) reflect self-organized adaptive strategies and needs vis-à-vis an ecology, it would be rather hard to provide more general classifications. Accordingly, we propose that the distinction between domain-specificity and domain-generality can be mitigated and reframed in terms of skillful adaptation.

### Adaptiveness as Novel and Functional World-Making

An understanding of musical creativity as an adaptive phenomenon integrating individual and collective dynamics, as we saw, places its visceral and participatory components at the heart of creative activity: this trades the focus on innate talent or divine gifts^8^ for a perspective that locates creative behavior and thought in openness, action, and uncertainty. Openness refers to the relational nature of adaptation, which is by definition organized around at least two elements (e.g., a performer and a composer, an organism and its niche, etc.) who participate in an ongoing dialogue; action here defines the capacity of agents to establish, transform, and extend such relationships in situations of online and offline (e.g., imaginative) interactions. Because of their openness and constantly shifting nature, the formed networks are subjects to continuous internal and external perturbations, involving processes and outcomes that are largely precarious and uncertain.

Before we approach this insight from a perspective inspired by enactive cognition, we note that recent work in neuroscience has increasingly explored the neurocognitive dynamics involved in prediction and minimization of uncertainty (see Friston, 2010). Here, a central idea is that rather than passively obtaining external information, the brain is thought to be able to estimate variances and uncertainties of sensory data by endlessly producing probabilistic models of the external world (see also Kolossa et al., 2015). Put simply, the view holds that the brain can be understood as a predictive machine that aims to minimize its prediction error (i.e., the difference between predicted and actual sensory events). This view, *prima facie*, appears to be unbridgeable with the study of more creativity-prone states, which on the contrary would include increased cognitive demands for novelty seeking and exploration. As Clark (2016) put it: “[t]he cognitive imperative of prediction error minimization, it is sometimes feared, is congenitally unable to accommodate such phenomena, offering instead a prescription for quietism, deliberate cognitive diminishment, (perhaps) even fatal inactivity!” (p. 262). As we read in the very next line, however, “this worry (though important) is multiply misguided” (Clark, 2016). In fact, living systems must continuously *act* to survive and flourish as situated agents. This crucially involves forming and dissolving couplings with the environment that are both context-sensitive as well as temporally and socially extended. Not only can prediction error be minimized by means of generating more accurate ways of sensing the future, but it can also be minimized when we exert causal influence on a given event, actively changing the latter to accommodate our prediction (see Friston et al., 2010).

For these organism–world relationships to be meaningful, functionality and novelty are essential: when interaction is not functional, as sometimes it happens during a musical performance, then a satisfactory overall product will not be likely achieved: musicians playing together may just not feel like they have a good “connection” with other performers or with the audience, resulting in unsatisfactory outcomes. Interaction also needs to include innovative features, otherwise its products will likely feature static, boring, or unexciting musical moments. Importantly, because we have suggested that interaction is pervasive of musicking even in the context of solo performance or composition, these empathic connections are not overshadowed when other participants are absent. In fact, in such cases, the lack of online interaction may be compensated by imaginative strategies (e.g., the composer thinking about how an audience will react to her own new piece), by a subtler “felt” presence of others, as observed in previously reported empirical studies, and by the sets of sedimented historical norms and social conventions that endow musical practices with their different recognizable statuses across cultures and communities. As we also saw earlier, the development and maintenance of such relationships require a constant negotiation of singular and plural dynamics: goals, actions, emotions, and musical ideas of lone agents may be skillfully transformed and re-adapted on the basis of newly established couplings and social needs. In brief, in their manifold experiences and manifestations, performing and composing music involve an interpenetration of individual and collective dynamics crystallized in cognitive relationships that are novel and functional or, indeed, *creative*. Interestingly, the same tension between internal and external factors observable at the basis of these forms of music-related organism–world couplings can be found in the set of homeostatic/allostatic self-regulatory activities living systems adopt to survive, develop intelligent behavior, and preserve their structural organization (i.e., to maintain the functional unity of the system, see Maturana and Varela, 1980). The recognition of a continuity between music and these bio-cognitive activities moves our discussion toward an analysis of wider creative properties.

Adaptiveness is a fundamental aspect of our life and rests at the core of enactive cognitive science, a framework that looks at mental activity as a process of organism–environment co-specification (Varela et al., 1991; Gallagher, 2017; Di Paolo et al., 2017). Enaction is an interdisciplinary school of thought that brings together scholarship in theoretical biology, artificial intelligence (AI), cognitive psychology, phenomenology, as well as neuroscience and consciousness studies, among others (see Stewart et al., 2010). At the heart of this approach, there is the conviction that living agents are best understood as autonomous, self-organized systems, which co-evolve (ontogenetically and phylogenetically) with their ambience *via* continuous sensorimotor loops^9^ (Varela et al., 1991; Thompson, 2005, 2007). Living beings are autonomous because they are organized to survive under precarious conditions by means of self-organization—the ability to separate themselves from the environment (Di Paolo, 2005, 2009). Importantly, this is a case of *differentiation*–not to be confused with *isolation* (De Jaegher et al., 2016): the living ecology in which organisms operate discloses perceptual, imaginative, and concrete action–opportunities for the re-organization and consolidation of the agent’s bio-cognitive domain. Indeed, on the basis of the latter’s morphological, behavioral, and cognitive complexity, various environmental affordances can be detected and acted upon^10^. As Fuchs (2018) notes, von Uexküll anticipated such insights when defining the organism–environment complementarity as a feedback loop of receptive and effective processes—a functional cycle that allows the animal to make sense of the world through evaluation and active engagement. Because evaluation and engagement allow the living system to predict threats, foresee resources, and optimize its natural inclination toward survival and well-being, the environment becomes existentially significant. The organism is thus understood as a “sense-maker” by enactivists because its being-in-the-world relies on the actualization of a meaningful perspective over its *umwelt*. A well-known passage by Evan Thompson offers a good example of how such a concerned perspective, or identity, rests upon a dynamical interplay between the organism’s autonomy, its meaning-making activity, and its entanglement with its ambience:

“Consider motile bacteria swimming uphill in a food gradient of sugar. These cells tumble about until they hit on an orientation that increases their exposure to sugar, at which point they swim forward, up-gradient, toward the zone of greatest sugar concentration. […]. [T]he way they move (tumbling or swimming forward) depends on what they sense, and what they sense depends on how they move. This sensorimotor loop both expresses and is subordinated to the cell’s autonomy. […] As a result, every sensorimotor interaction and every discriminable feature of the environment embodies or reflects the bacterial perspective. Thus, although sucrose is a real and present condition of the physicochemical environment, its status as food is not. That sucrose is a nutrient is not intrinsic to the sucrose molecule, but is a relational feature, linked to the bacterium’s metabolism. Sucrose has significance or value as food, but only in the milieu that the organism itself enacts. Thus, thanks to the organism’s autonomy, its niche has a ‘surplus of significance’ compared with the physicochemical environment”

(Thompson, 2005, p. 418, quoted in Villalobos and Ward, 2015).

Autonomous agents, such as bacteria, human beings, or other animals, skillfully adapt to internal and external perturbations by bringing forth (i.e., *enacting*) a world (Varela et al., 1991; Froese and Di Paolo, 2011; Di Paolo et al., 2017; De Jesus, 2018). Enactivists argue that mental life originates in such a self-organized, world-making activity (Weber and Varela, 2002; see also van der Schyff, 2015; Schiavio and van der Schyff, 2018 for music-related insights). As we saw, there is an important topological tension between this characterization of the organism’s individuality and its openness to its surroundings (Di Paolo and Thompson, 2014). Living agents realize themselves and develop their identity through their metabolic activity, whose operating structures must be separated from external perturbations. At the same time, organisms regulate this activity through exchanges of energy and information with the world they inhabit, giving rise to an adaptive loop. Notably, “[t]his regulation […] does not mechanically react to limited sets of occurring stimuli on the basis of the statistical repetition of previous experiences, but also flexibly prioritizes between novel contingencies based on their contextual relevance for the survival of the organism, anticipating the incoming changes” (Cappuccio and Froese, 2014, p. 6).

Living systems, therefore, must create organism–world couplings that are *functional* and conducive to survival. To do so, these couplings often need to be *innovative*: the constant reframing of internal dispositions and relational dynamics involves risk-taking and uncertainty, which can in turn result in reward. With this is mind, categories, such as curiosity, exploration, as well as novelty-seeking, may further motivate the enactment of a world that is tailored for action, as engagement with ambiguous sensory information will ultimately produce reward^11^. As stated earlier, however, this not only concerns how precise our “interoceptive (bodily), exteroceptive (external) and proprioceptive (motoric) sensory predictions” (Ondobaka, 2017, p. 1332) may be; rather, the minimization of prediction error also involves our embodied capacity to generate experience through action, thereby fostering the creation of new regularities (see Schmidhuber, 2006). Conversely, stationary situations featuring low levels of uncertainty will likely give rise to less functional organism–world couplings, as curiosity rewards are hindered. It should also be noted that when couplings stabilize, there might always be some perturbatory condition that would make the unfolding interaction lean toward particular action-tendencies, disrupting the optimal balance that was initially created. Constant adjustments and control are thus needed to support and maintain the precarious equilibrium between living beings and their niche, recalibrate predictions, and produce efficient solutions for task-specific and open-ended problems. Such adjustments might be described in terms of actions, emotions, sensorimotor schemas, motivations, as well as (context-specific or general) social adaptations. For example, novel behavioral configurations may be developed and re-adapted to better explore one’s peripersonal space and address physiological and psychological needs emerged under new ecological conditions. In the following lines, we explore in more detail how such insights may be relevant to creativity research, with a special focus on the issue of domain-generality and domain-specificity.

### Enacting Creativity

The novel and functional adaptations at the basis of the capacity to establish meaningful couplings with the world allow the living system to achieve a certain goal—ranging from the realization of a stable thermodynamic equilibrium with the environment in unicellular organisms to the participation in artistic events for more complex animals like us. For very basic forms of life, this ongoing bidirectional dependency may only relate to a quest for nutrition and the different adaptations this entails; but for more sophisticated beings, such as humans, needs and motivations span different situations and experiences and may include art and music (van der Schyff and Schiavio, 2017; see also Dissanayake, 1988, 1995; Kaufman, 2020). In both music-specific contexts and general bio-cognitive domains, it is suggested that the tension between operational closure and material openness is overcome when a veil of significance is casted upon the environment: this allows living systems to anticipate or address perturbations and take care of and restore their internal metabolic balance as well as their state of equilibrium with their ecology. By doing so, they *enact* their identity, thereby combining local (endogenous) and global (world-involving) contingencies into a newly structured unity. In the following quote, jazz improvisation is taken as an example to describe such bio-cognitive dynamics:

“The organism’s environment is a world of elements that matter to the organism, as assisting or threatening the latter’s self-maintenance. So the environment is not a neutral, exterior world but a world already interpreted as an array of self-generated significances. It is perhaps not too far a stretch to say that the continual unfolding of the process of an organism’s meaning-making encounter with its environment is like an improvising jazz musician generating musical responses that make sense in the context of her fellow players’ (and her own) previous musical ‘moves”’

(Torrance and Schumann, 2019).

Here, the environment is not conceptualized as a pre-given structure “out there” displaying fixed properties and regularities that can be objectively assessed. Rather, it is first and foremost understood as an ongoing network of organism-specific relationships with significance, value, and affordative opportunities that differ across domains and contexts. In other words, “the environment is not a structure imposed on living beings from outside but is in fact a creation of those beings. The environment is not an autonomous process but a reflection of the biology of the species” (Lewontin, 1983, p. 99). Through the enactment of their unique perspectives, agents become meaning-makers who dynamically co-evolve with the world they inhabit. In musical contexts, the environment affords more than changing extant behaviors or regulating metabolic functions: the creation of a musical niche *via* acts of musicking, as we saw, includes online and offline forms of social experience developed through face-to-face situations, or through explicitly imaginative or “felt” dynamics. It is within this adaptive interplay that a concerned, musical perspective is brought forth into the world:

“traditionally, music composition and performance, have been understood as a realization of preconceived musical structures that through the perceptual and cognitive processes of replication or invention are presented either in real time (as performance) or over an extended period of time (as composition). Yet the nature of musical creativity may suggest further emotional and musical representations of specific, freely associated experiences constructed by the composer or performer. […] Thus, musical creativity, can be best defined as a form of self-realization—a discovery and manifestation of the existence of an authentic self”

(Nagy, 2017, p. 73).

This “authentic self,” we suggest, escapes individualistic descriptions as it involves both singular and plural dynamics (see Kyselo, 2014 for an accurate analysis of the “enactive self,” which emphasizes the role of social interaction). These dynamics are constructed through forms of direct interactions (as when making music together), or through other world-involving engagements (e.g., the constructed norms and conventions to which musicians playing alone intuitively adapt to and transform). But because needs and goals must also reflect the operational closure of the system, the individual components involved in establishing and maintaining the described organism–environment loops are not dissolved; they are enacted in a recursive cycle of skillful adaptations, showing once again how “the boundaries that distinguish self from other, instead of being fixed and hard won, are under constant renegotiation” (Valencia and Froese, 2020).

This insight prompts us to re-assess the polarization between domain-generality and domain-specificity that often frames research and theory in the field of creativity. Creative thoughts and actions that are relevant to a given domain, we suggest, rely on a more general tendency of living systems: the capacity to establish meaningful, novel, and functional relationships with the world they co-evolve with. Accordingly, while different creative artifacts may be produced in response to specific demands, the processes underlying creative production reflect a common bio-cognitive core. But since the working of the latter depends on a continuous interaction between living systems (with their own perspectives, identities, experiences, needs, etc.), and their milieux, it exhibits a self-organized variability that can be hardly articulated in more general terms. In other words, we argue that creative effort entails a range of uniquely developed, specific adaptations, which continuously transform the couplings between an organism and its niche. As these couplings are subject to never-ending feed-back and feed-forward loops involving local and global dynamics, their states are always shifting and transitory. We thus maintain that empirical approaches and theoretical insights that posit a strong separation between domain-specificity and domain-generality may not be enough to capture the wide spectrum of situated activities involved in creative cognition. Instead, we propose that an understanding of creativity as a skillful organism–world adaptation offers a way forward, allowing scholars to better assess the continuous interplay of micro- and macro-scale factors in creative effort. For example, one might examine how broader social, cultural, and ecological dynamics contribute to rapid modifications of creative choices in a given context, and how differences in specific creative activities across domains may affect in real-time more general organism–world couplings (e.g., emotional regulation, social cognition, etc.). Notably, this focus on skillful adaptation allows us to refer to creativity not as a quality that one has or not, but rather as a mode of engagement with the world that one continuously cultivates and brings into the daylight of experience through situated action.

Before concluding, we should note that insights from enactive cognitive science have inspired the development of computational models of creativity in AI (Froese and Ziemke, 2009; Guckelsberger et al., 2017), as well as analyses that focus on the continuity between mindfulness, skilled proficiency, skill acquisition, and the creative activity of improvisers and musical learners (e.g., Schiavio and van der Schyff, 2018; van der Schyff et al., 2018; Torrance and Schumann, 2019). An understanding of musical creativity as adaptation has been also proposed by Reybrouck (2006), who draws a fascinating parallel between the process of dealing with music (described as a skillful form of coping with the sonic world) and epistemic control systems. The latter, in cybernetics, denotes any adaptive device that displays a closed operating loop allowing a constant adjustment to external disturbances. The individual (or the “music user,” in his terms) is thus seen by Reybrouck as an adaptive device able to modify its relations with the world by evaluating perceptual primitives and acting upon them consistently. This would reduce external perturbations and induce novel compensatory strategies in the user (i) to alter and expand its perceptual repertoire and (ii) to actively manipulate the world and produce novel musical artifacts. The idea that creativity emerges in the flexible interplay between evaluating and controlling the environment resonates well with the perspective outlined in this paper and aligns with recent views in ecological dynamics that conceive of creativity as a function of the organism–world perceptual attunement (e.g., Araújo et al., 2017; Kimmel, 2017, 2019). This also echoes the description of *creative ecology* offered by Howkins, who states that “creativity is […] a rich mix of ecological factors, primarily diversity, change, learning, and adaptation. It exists only where the ecology permits and it flourishes through adaptive efficiency” (quoted in Barrett, 2012, p. 213). These accounts are particularly well suited to address the motor productivity that characterizes most joint practices (e.g., dance, team-sports, collaborative music-making, etc.; see Gruber, 1989; Hristovski et al., 2011, 2012), emphasizing once again how patterns of adaptive engagement can dynamically transform the experience of the here-and-now and produce variabilities that emerge in longer time-scales.

## Conclusion and Future Directions

During an interview^12^ broadcasted in 1969, Italian composer and conductor Bruno Maderna was asked whether he would conceive of music as an intellectual operation or as a praxis guided by more primordial (e.g., emotional) needs. His answer was that music in general (and musical theater in particular) is best understood as a “social fact,” a “necessity,” and “a mirror of the relationship between society and the individual.” Similar views of music and musical practices have been explored in various ways by scholars working in the context of ethnomusicology and social sciences (e.g., Turino, 2008), music education (e.g., van der Schyff et al., 2016), and evolutionary musicology (e.g., Cross, 2001). Moving from these insights, in this paper, we have argued that creative cognition (in music and beyond) may be understood as an adaptive phenomenon that originates in a primordial, and necessary, sense-making activity—a bio-cognitive inclination to create, transform, and maintain viable relationships with the world. As we saw, this perspective helps mitigate two dichotomies that often drive research and theory in the field: that between individuality and collectivity and that between domain-generality and domain-specificity.

With regard to the former dichotomy, we have discussed how composers and performers often establish meaningful musical connections with others during moments of online and offline interactions, that is, even in cases where the social “other” is physically absent. As Small put it, “any ‘artistic’ performance, if one examines it with attention, will show itself to involve more than the art which is ostensibly occupied” (Small, 1998, p. 109). And this “more” might be accounted for by considering the interpersonal and cultural contingencies that variously take part in solo musical activity. Accordingly, we have discussed a variety of cases of creative solitary musicking and explored their hidden “plural” and adaptive components. Our examples included (sometimes overlapping) experiences of music composition and performance, ranging from explicitly collaborative activities to the construction of a virtual presence of other performers, composers, or audience members. Are these cases of individual or collective creativity? At the end, the two prove inseparable because aspects that pertain to the most intimate sphere of our individuality (agency, volitions, proclivities, emotions, etc.) are ultimately co-constituted by exogenous factors, and it is in this organism–world co-evolution that creative thinking and doing flourish (see also van der Schyff and Schiavio, in press).

To address the second dichotomy (i.e., domain-general vs. domain-specific creativity), we moved to another scholarly domain, that of enactive cognitive science. By exploring the core tenets of this approach, we have discussed how mental life (and not only creative cognition) can be conceived of as a process whereby agents actively shape and at the same time adapt to the environment in which they are situated. This, as we saw, gives rise to open-ended adjustments in thought and action, allowing agents to creatively (re-)establish, assemble, and decompose different organism–world relationships. We say *creatively*, because these relationships exhibit two properties–*novelty* and *functionality*–that are defining of creative activity and that many scholars would deem creative. Indeed, for such relationships to be “successful,” they must continuously renew themselves without moving too far from the contextual landscape from which they originate. To better account for this overlap between creative cognition and mental life, in which individual and ecological factors are constantly negotiated to produce meaningful organism–world couplings, we have reframed the issue of domain-specificity and domain-generality in different terms. That is, rather than understanding domain-generality and domain-specificity as contrasting views that inform empirical practice and theory in one way or another, we have laid down the basis of a conceptual framework that sees creativity as a process of *skillful adaptation*. Here, general principles pertaining to the bio-cognitive organization of living systems (i.e., the capacity to form novel and functional relationships with the world) and specificities of each individual agent (i.e., their unique identity) are thought to be systematically combined in the creative act.

The recognition of a continuous integration of individual, collective, domain-general, and domain-specific creative factors that emerges from our hybrid account can open up fascinating possibilities for future experimental and theoretical work, helping formulate precise empirical questions and fostering interdisciplinary analyses. For an example, we may consider how, in order to produce various compensatory actions to keep their musicking “alive” and pulsating, musicians often decenter their agency, producing patterns of reciprocal exchanges that stabilize and destabilize their activity on the spot (see Ryan and Schiavio, 2019). Here, openings and constraints functional to creative activity are shared between individuals, groups, and ecological variabilities, suggesting that each performer must always negotiate singular and plural dynamics and continuously (re-)generate a range of novel couplings with his or her niche. These couplings, as we have argued, not only involve immediate interactions with co-performers and audience (e.g., to monitor the functionality of precise contextual online adaptations) but also extend to include larger social dynamics (e.g., to situate their musicking into an appropriate context). To better capture this point, we may use the following quote from Orth et al. (2017), with an important addition (in italic):

“actions are considered as emergent in the temporary couplings formed among the individual and the environment […]. Importantly, these couplings are not uniquely determined by the individual’s characteristics, but in unity with environmental and task constraints. These constraints define the space within which the movement system can act, placing boundaries on the movement solutions available […]. From this perspective, creative motor actions are as much a function of the individual, as the task and [*the broader cultural, social, and historical*] environment”

(Orth et al., 2017).

In musical terms, creativity here would concern how musicians might intentionally “play” with the continuous integration of such local and global dynamics, making each performance unique by fluidly crisscrossing the boundaries between control, risk-taking, contextuality, and spontaneity (see also Berkowitz, 2010; Schiavio and Kimmel, under review; Wopereis et al., 2013). This could help performers generate convincing outcomes that are at the same time original and stylistically coherent, by navigating the range of vicissitudes and adaptations (e.g., emotion, proclivity, empathy, etc.) that shape their coupling with the world in the (precarious, uncertain) here-and-now of creative effort.

Such insights may also be relevant for the neuroscientific community when they can contribute to develop precise research questions and testable hypotheses. An example involves the role played by the sense of agency in creative performance. A recent study by Beyer et al. (2018) demonstrates that participants engaged in social trials (i.e., where decisions to act or not to act depend on another individual) exhibit increased activation of the bilateral temporo-parietal junction (TPJ), precuneus, and middle frontal gyrus when compared with non-social situations. In musical contexts, it has been shown that TPJ activity normally decreases when experts improvise music, whereas it does not change when novices perform the same task (Berkowitz and Ansari, 2010). This suggests that while TPJ may be “naturally” involved in the creation of novel musical outcomes, experts may have inhibited its activation through years of training, as they have been voluntarily engaging with processes involving more self-focused attention. However, while TPJ is modulated by social contexts, it is not affected by the sense of agency. The activity of the precuneus (as demonstrated in the same study by Beyer et al., 2018) tends instead to increase in social conditions and positively correlates with decreased sense of agency. Because our analysis suggests that solo creative activities involve a good deal of intersubjectivity, and because contexts featuring the presence of others are often associated with a reduction in the feeling of being in control of our own actions (see e.g., Sidarus et al., 2020), we would expect that drops in an individual’s sense of agency can be observed in subjects performing a creative task by themselves. And as decreased sense of agency is also correlated to the activation of the precuneus in the brain, the prediction can be made that particularly significant moments of creativity (e.g., achieved during solo music improvisation) would involve systematic associations between drops in the sense of agency (e.g., reported verbally) and increased activity of the precuneus (e.g., revealed by fMRI). We have already considered qualitative insights that point to this direction, with verbal descriptions offered by novice and expert musicians highlighting feelings of shared corporeality (see again the “Performing Music” section). It would be thus very interesting to see whether the possible empirical scenario we have outlined would give rise to such results in a sample of both experts and novices. The same experimental setting could also be extended to include and compare other (i.e., non-musical) domains, as the activity of the precuneus has been already positively associated with divergent thinking more generally (see e.g., Benedek et al., 2014b; Jauk et al., 2015).

This last example illustrates well how the recognition of a profound overlap between individuality and collectivity, as found in musical contexts, may stimulate the development of conceptual and experimental tools in other areas. This could help us better navigate the differences between the various dimensions of musical and non-musical creativities, observe in more detail their singular and social components, and describe with increased accuracy the network of adaptations and adjustments at the basis of creative effort, looking beyond existing dichotomies. In conclusion, we hope that researchers investigating the psychology and the neuroscience of creativity, the working of the musical mind, and enactive cognition, could join forces to further develop the insights presented here, providing empirical validations of specific claims and offering novel theoretical resources for research and theory.

## Data Availability Statement

No experimental data was generated for the present article.

## Author Contributions

AS and MB co-wrote and co-edited the manuscript. They have made a substantial, direct and intellectual contribution to the work, and approved it for publication.

## Conflict of Interest

The authors declare that the research was conducted in the absence of any commercial or financial relationships that could be construed as a potential conflict of interest.

## Footnotes

1 As we will see in “The Ubiquity of Skillful Adaptation” section, there is an important sense in which basic bio-cognitive properties of living systems can be described in terms of organism–world co-specification, thereby including social dynamics into individual processes.

2 Methodologically, analyses of this joint activity often rely on historically informed research, in which correspondence letters between composers and performers are examined and put into context. Examples can be found in the missives exchanged by renewed classical guitarist Andrés Segovia (1893–1988) and the composers (e.g., Manuel Maria Ponce, Mario Castelnuovo-Tedesco, etc.) who did heed his call to write new pieces for guitar to make the latter’s repertoire blossom. “Many of these composers, […] were not familiar with the classical guitar, and it was necessary for Segovia to collaborate with them in order to make the music playable” (Knapp, 2011, p. 2).

3 This is a “a measurable concept of causality or directed influence for time series data, defined using predictability and temporal precedence” (Roebroeck, 2015).

4 This echoes insights from Eric Clarke, who suggests that music performance can be considered as “the construction and articulation of musical meaning, in which cerebral, bodily, social, and historical attributes of a performer all converge, and if we choose to regard this convergence as an expression of the performer’s mind, then we must remember that the mind is neither driving the body nor confined within the head” (Clarke, 2002, p. 68–69; see also Leman, 2008).

5 For example, empirical work by Repp (1997) suggests that the generally preferred style of rubato is basically the average of what most performers are doing today. This is presumably different from rubato at the time the work was composed (see also Parncutt, 2003; Bisesi et al., 2019).

6 This suggestion aligns well with recent advances in “second person” cognitive neuroscience that place major emphasis on the organism–environment coupling and the interactive nature of human cognition and experience (see e.g., Hari and Kujala, 2009; Dumas, 2011; Redcay and Schilbach, 2019).

7 According to Wiggings, such a gamut “allows us to make distinctions between conscious creation in the deliberate planning of a formalist composer, the semi-spontaneous but cooperative and partly planned creation of the jazz improviser in a trio, and the entirely spontaneous whistling in the street of the same people that Schoenberg famously hoped and failed to convince of his 12-note ‘tunes”’ (Wiggins, 2012).

8 “If we take seriously the dictionary definition of creation, ‘to bring into being or form out of nothing’, creativity seems to be not only beyond any scientific understanding, but even impossible. It is hardly surprising, then, that some people have ‘explained’ it in terms of divine inspiration, and many others in terms of some romantic intuition, or insight” (Boden, 1998, p. 15). A good example of this can be found in the mystical aura that often surrounds Western classical composers and performers whose creative genius has been traditionally associated with innate talent (see e.g., Montuori and Purser, 1995). This stereotypical view has perpetuated, and legitimized, a still pervasive characterization of “great” Western composers–“since the rest do not make into the myths” (Cook, 2018, p. 73)–as heroic creators who can channel into music divine inspiration and knowledge (see Cook, 2006; Hill, 2018).

9 “The organism is understood here to play an active role in shaping the environment it *coevolves with*—its activities feedback into and alter the selective pressures of the environmental niche. This, in turn, affects the development of the organism, resulting in a co-evolutionary cycle that proceeds in an ongoing way” (van der Schyff et al., 2018).

10 This last aspect recalls classic insights developed in ecological psychology, and one could thus wonder how the latter framework relates to enactive cognition. Scholars working in ecological psychology draw their influence from James Gibson and his approach to visual perception, whereas advocates of enactive cognitive science usually individuate Francisco Varela as its chief representative. As recently pointed out by Baggs and Chemero (2018), these two school of thoughts have been often considered at odds with each other, advocating seemingly opposite starting assumptions: “[e]cological psychologists have traditionally asserted a commitment to realism, while enactivism was initially developed within a constructivist, and therefore anti-realist, framework” (Baggs and Chemero, 2018). In fact, this “deep contrast” is most evident in early versions of both doctrines and has been attenuated in recent years (see Chemero, 2009).

11 Recent work has showed that fluctuations in uncertainty are important aspects of aesthetic appreciation and emotional experience of music (Koelsch, 2014; Daikoku, 2019).

12 The entire interview can be watched at https://www.youtube.com/watch?v=5AxNcusxShQ.
